# BAP1 expression is prognostic in breast and uveal melanoma but not colon cancer and is highly positively correlated with RBM15B and USP19

**DOI:** 10.1371/journal.pone.0211507

**Published:** 2019-02-04

**Authors:** Leili Shahriyari, Mohamed Abdel-Rahman, Colleen Cebulla

**Affiliations:** 1 Department of Mathematics, University of Texas Arlington, Arlington, Texas, United States of America; 2 Division of Human Genetics, Department of Internal Medicine, The Ohio State University, Columbus, Ohio, United States of America; 3 Havener Eye Institute, Department of Ophthalmology and Visual Science, The Ohio State University Wexner Medical Center, Columbus, Ohio, United States of America; University of South Alabama Mitchell Cancer Institute, UNITED STATES

## Abstract

*BAP1* is a tumor suppressor gene important to the development and prognosis of many cancers, especially uveal melanoma (UM). Its role in more common cancers such as breast and colon cancer is largely unknown. We collected the transcriptome profiling data sets from the TCGA uveal melanoma (TCGA-UVM), breast cancer (TCGA-BRCA), and colon cancer (TCGA-COAD) projects to analyze the expression of *BAP1*. We found that patients with UM and breast cancer, but not colon cancer, who died had a lower level of *BAP1* gene expression compared to surviving patients. Importantly, in breast cancer patients, the lowest *BAP1* expression levels corresponded to the dead young patients (age at diagnosis < 46). Since the number of cases in TCGA-BRCA was much higher than TCGA-UVM, we obtained highly correlated genes with *BAP1* in invasive breast carcinomas. Then, we tested if these genes are also highly correlated with *BAP1* in UM and colon cancer. We found that *BAP1* is highly positively correlated with *RBM15B* and *USP19* expression in invasive breast carcinoma, UM, and colon adenocarcinoma. All three genes are located in close proximity on the 3p21 tumor suppressor region that is commonly altered in many cancers.

## Introduction

Both somatic and germline mutations in the BRCA1 associated protein-1 (*BAP1*) gene have been observed in many cancers. *BAP1* tumor predisposition syndrome (*BAP1* -TPDS) OMIM #614327 is associated with an increased risk for uveal melanoma (UM), malignant mesothelioma (MMe), cutaneous melanoma (CM), clear cell renal cell carcinoma (RCC) and potentially other cancers such as breast cancer [1–3].

UM is the most common and earliest presenting cancer in the BAP1-TPDS [1–3]. In addition, UM has the highest frequency of pathogenic somatic mutations in *BAP1* [4, 5]. Although mutations in *BAP1* are strongly associated with metastatic UMs (84%), it is not frequent in non-metastatic tumors (4%) [4]. Moreover, germline *BAP1* mutations are associated with more aggressive UM [6]. *BAP1* also plays a prognostic role in other cancers such as RCC and cholangiocarcinoma [7–9].

Although it is known that *BAP1* is a tumor suppressor gene important to the development and prognosis of many cancers, especially UM, its role in more common cancers including breast and colon is largely unexplored.

Here, we collected RNA-seq data sets from TCGA-BRCA, -UVM, and -COAD projects at Genomic Data Commons Data Portal, and we employed computational techniques to investigate the role of *BAP1* in UM and two common cancers, breast and colon. Furthermore, we evaluated how patient age, sex, and race may be associated with *BAP1* gene expression and patient survival with these cancers. We hypothesized that *BAP1* gene expression could be a potential signature for aggressive tumors. In addition we identified genes with expression that is highly correlated with *BAP1*. To determine the genes that may be critical to *BAP1* functional pathways, we used the UM data set which is known to have a high level of *BAP1* alteration to identify candidates, which were validated in the breast and colon cancer data sets.

We analyzed the data sets by applying two slightly different normalization methods. In the first method, we evaluated all genes and obtained z-scores to normalize the data. In the second method, we excluded the genes with values of zero for all patients in the data set, and then we obtained z-scores. Importantly, the results were independent of these two normalization strategies.

## Results

### Dead patients had low *BAP1* expression compared to survivors in breast cancer and UM but not colon cancer

**Fig 1:**
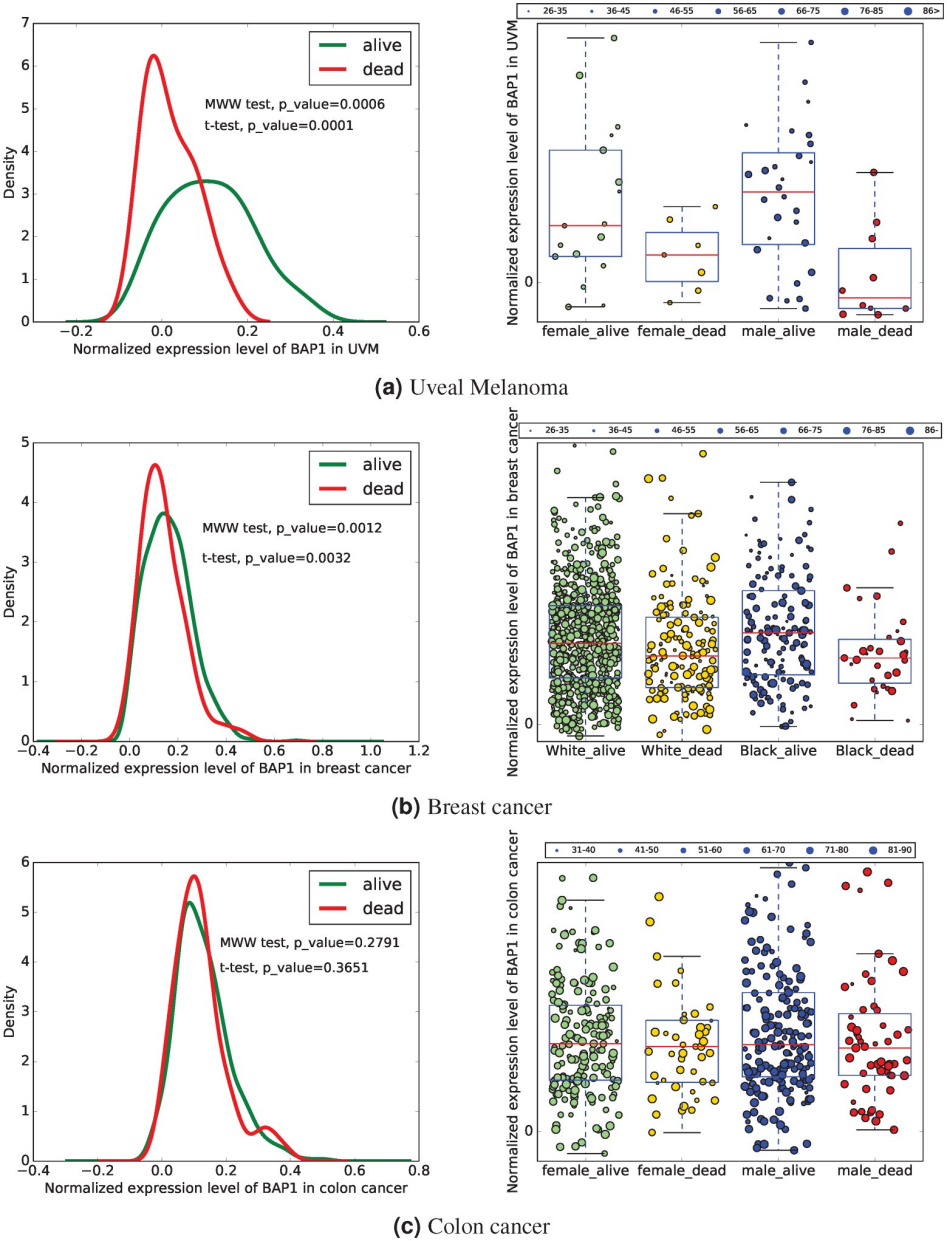
*BAP1* expression. The left and right sub-figures respectively represent the density function and the values of *BAP1* after normalizing the data set excluding genes with zero values. The size of circles represents the age at diagnosis.

We divided UM patients in four sub-categories based on their gender and vital status: female-alive, female-dead, male-alive, and male-dead (Table 1). We also divided patients into sub-groups based on their age at diagnosis (grouped into intervals of 10 years starting from 26, which was the age of the youngest patient). The left panel of Fig 1(a) shows the normalized level of *BAP1* for these patients, and the right sub-figure represent the density function for the values of *BAP1* in dead and alive patients after excluding genes with zero values and normalizing the data set. This figure indicates that the expression level of *BAP1* is lower in dead patients compared to the survivors regardless of their gender.

**Table 1:** Data set for UVM.

categories	number of cases	number of files
female	40	40
male	48	48
sub-groups	number of cases	number of files
female-alive	31	31
female-dead	9	9
male-alive	32	32
male-dead	16	16

We also divided breast cancer female patients in four sub-categories based on their race and vital status: black-alive, black-dead, white-alive, and white-dead (Table 2). The left sub-figure of Fig 1(b) shows the normalized (z-score) value of *BAP1* for survival categories of black and white female breast cancer patients, and the right sub-figure compares the *BAP1* expression in survivors with the *BAP1* expression in dead patients. In this figure, we also divided breast cancer patients into sub-groups based on their age at diagnosis (grouped into intervals of 10 years starting from 26, which was the age of the youngest patient). We found that dead patients, regardless of their race, have a lower level of *BAP1* expression compared with living patients. Importantly, the smallest level of *BAP1* corresponds to the young (age at diagnosis < 46 years old) patients who died.

**Table 2:** Data set for BRCA.

categories	number of cases	number of files
female	1084	1208
male	12	13
sub-groups-females	number of cases	number of files
black-alive	151	156
black-dead	29	31
white-alive	636	705
white-dead	112	159

Additionally, we investigated the expression level of *BAP1* in colon cancer. We divided patients with colon cancer in four sub-categories based on their gender and vital status: female-alive, female-dead, male-alive, and male-dead (Table 3). The left sub-figure of Fig 1(c) shows the normalized level of *BAP1* for these patients, and the right sub-figure represents the difference between the *BAP1* expression in dead and alive patients. We also divided colon cancer patients into sub-groups based on their age at diagnosis (grouped into intervals of 10 years starting from 31, which was the age of the youngest patient). This figure indicates that the expression level of *BAP1* in patients with colon cancer is not correlated with vital status or gender.

**Table 3:** Data set for COAD.

categories	number of cases	number of files
female	214	224
male	240	252
sub-groups	number of cases	number of files
female-alive	168	177
female-dead	46	47
male-alive	184	196
male-dead	56	56

### *BAP1* is highly positively correlated with *RBM15B* and *USP19*

**Fig 2:**
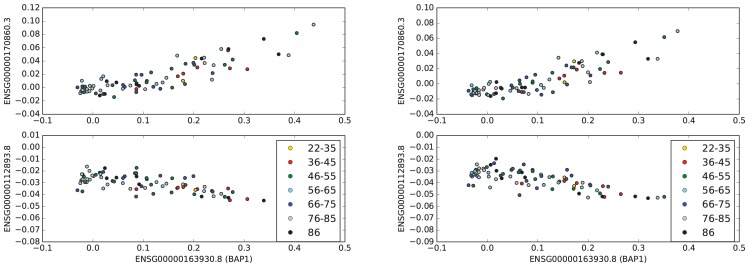
Highly correlated genes with *BAP1* in UVM. The top sub-figure shows the scatter plot of the values of *BAP1* and the gene, which has the maximum positive correlation with *BAP1* in the entire UVM data set. The bottom sub-figure shows the scatter plot of the values of *BAP1* and the gene, which has the maximum negative correlation with *BAP1* in all patients with UVM. The right panel shows the exact same scatter plot as the left panel using n-normalization.

We found the genes that were highly correlated (absolute value of correlation coefficient > 0.8) with *BAP1* in the entire TCGA-UVM dataset. Although there were 24 genes with a high positive correlation with *BAP1*, there were no genes with a high negative correlation with *BAP1*. Table 4 shows the genes that are highly positively correlated with *BAP1* in the entire data set of UM. Top panels (and bottom panels) of Fig 2 show the scatter plot of values of *BAP1* and the genes with the highest positive and negative correlation coefficients with *BAP1* in UM.

**Table 4:** Highly correlated genes with *BAP1* in the entire UVM dataset.

transcript (gene)	(cor, p-value)	location
ENSG00000114316.11 (USP4)	(0.84,1.4e-24)	3p21.31
ENSG00000177352.9 (CCDC71)	(0.832,1.0e-23)	3p21.31
ENSG00000259956.1 (RBM15B)	(0.838,2.4e-24)	3p21.2
ENSG00000178149.15 (DALRD3)	(0.808,1.8e-21)	3p21.31
ENSG00000161202.16 (DVL3)	(0.854,4.1e-26)	3q27.1
ENSG00000114767.6 (RRP9)	(0.824,5.7e-23)	3p21.2
ENSG00000114650.17 (SCAP)	(0.84,1.3e-24)	3p21.31
ENSG00000171135.12 (JAGN1)	(0.802,6.5e-21)	3p25.3
ENSG00000172046.17 (USP19)	(0.838,2.2e-24)	3p21.31
ENSG00000175792.10 (RUVBL1)	(0.806,2.7e-21)	3q21.3
ENSG00000164062.11 (APEH)	(0.842,8.0e-25)	3p21.31
ENSG00000183624.12 (HMCES)	(0.804,4.2e-21)	3q21.3
ENSG00000176095.10 (IP6K1)	(0.824,5.9e-23)	3p21.31
ENSG00000114738.9 (MAPKAPK3)	(0.815,4.5e-22)	3p21.2
ENSG00000163932.12 (PRKCD)	(0.824,6.3e-23)	3p21.1
ENSG00000187091.12 (PLCD1)	(0.816,3.7e-22)	3p22.2
ENSG00000163719.17 (MTMR14)	(0.848,2.1e-25)	3p25.3
ENSG00000213672.6 (NCKIPSD)	(0.824,6.4e-23)	3p21.31
ENSG00000114544.14 (SLC41A3)	(0.819,1.8e-22)	3q21.3
ENSG00000270170.1 (NCBP2-AS2)	(0.851,9.2e-26)	3q29
ENSG00000144659.9 (SLC25A38)	(0.857,1.8e-26)	3p22.1
ENSG00000170860.3 (LSM3)	(0.876,6.3e-29)	3p25.1
ENSG00000132153.13 (DHX30)	(0.824,6.9e-23)	3p21.31
ENSG00000163930.8 (BAP1)	(1.0,0.0)	3p21.1
ENSG00000145191.10 (EIF2B5)	(0.822,9.8e-23)	3q27.1

**Fig 3:**
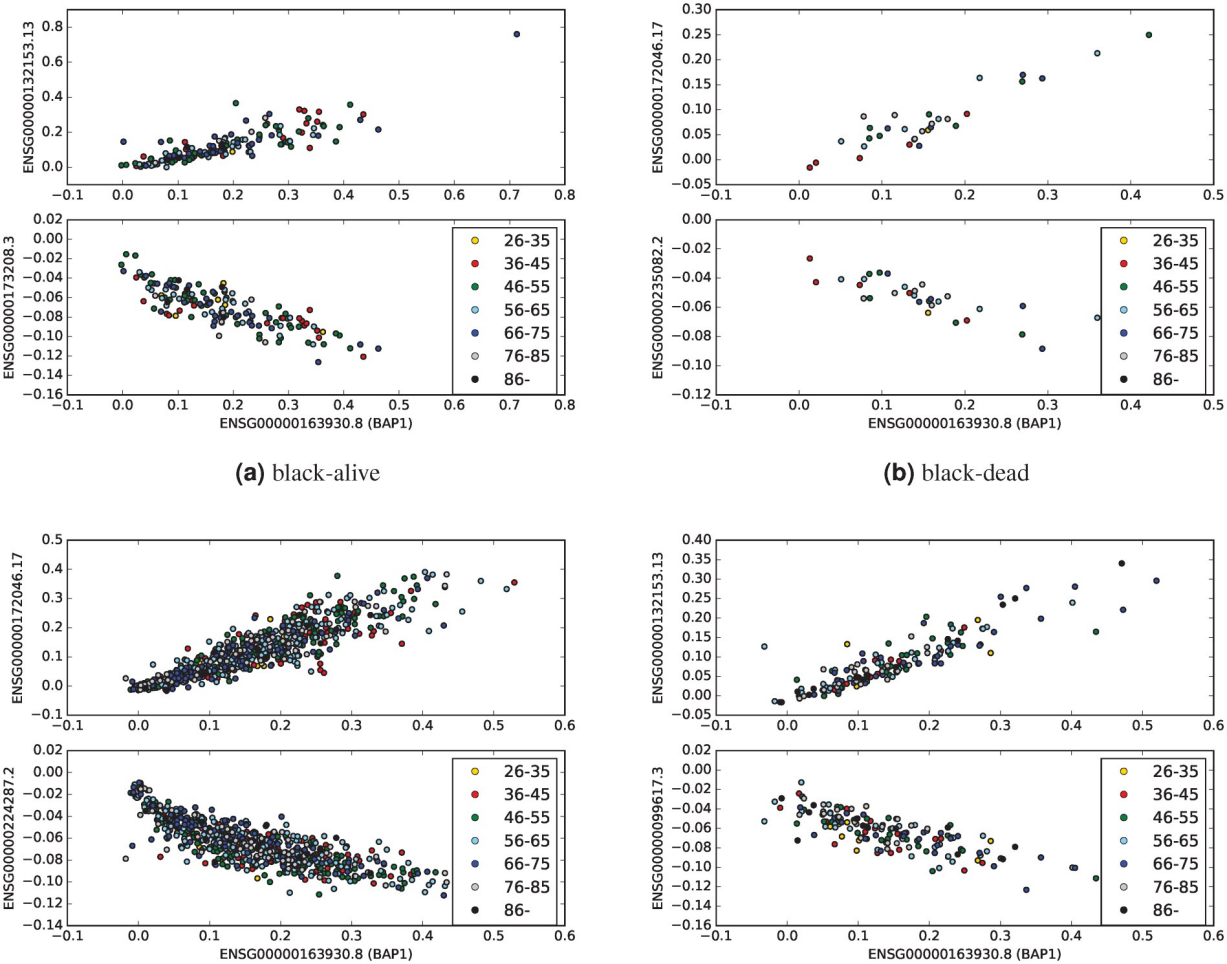
Highly correlated genes with *BAP1* in BRCA sub-categories. In each panel, the top sub-figures show the scatter plot of the values of *BAP1* and the gene, which has the maximum positive correlation with *BAP1* in the given BRCA sub-category. The bottom sub-figures shows the scatter plot of the values of *BAP1* and the gene, which has the maximum negative correlation with *BAP1* in the given sub-category.

In patients with breast invasive carcinomas, *BAP1* is highly positively correlated with NSG00000132153.13 (*DHX30*), ENSG00000259956.1 (*RBM15B*), and ENSG00000172046.17 (*USP19*), in all four categories (Table 5). Importantly, all these three genes were also highly positive correlated with *BAP1* in UM (Table 1). *DHX30* has the highest positive correlation with *BAP1* in black-alive and white-dead categories, while *USP19* has the maximum positive correlation with *BAP1* in black-dead and white-alive breast cancer categories. Fig 3 presents scatter plots of values of *BAP1* and the genes with the highest positive and negative correlation coefficient with *BAP1* in all four breast patients categories.

**Table 5:** Highly correlated genes with *BAP1* in BRCA data set.

transcript (gene)	black-alive (cor, p-value)	black-dead (cor, p-value)	white-alive (cor, p-value)	white-dead (cor, p-value)	all-data (cor, p-value)	location
ENSG00000132153.13 (DHX30)	(0.839,8.1e-42)	(0.905,2.8e-12)	(0.87,6.4e-219)	(0.895,1.3e-56)	(0.87,1.3e-321)	3p21.31
ENSG00000163930.8 (*BAP1*)	(1.0,0.0e+00)	(1.0,0.0e+00)	(1.0,0.0e+00)	(1.0,0.0e+00)	(1.0,0.00e+00)	3p21.1
ENSG00000198218.9 (QRICH1)	(0.732,6.0e-27)	(0.827,9.8e-09)	(0.853,2.3e-201)	(0.841,1.6e-43)	(0.82,8.7e-255)	3p21.31
ENSG00000126062.3 (TMEM115)	(0.726,2.6e-26)	(0.806,4.6e-08)	(0.828,6.7e-180)	(0.848,8.1e-45)	(0.81,1.9e-248)	3p21.31
ENSG00000259956.1 (*RBM15B*)	(0.834,8.0e-41)	(0.911,1.1e-12)	(0.86,1.6e-208)	(0.86,1.5e-48)	(0.85,4.6e-302)	3p21.2
ENSG00000178252.16 (WDR6)	(0.752,4.1e-29)	(0.896,9.2e-12)	(0.848,3.2e-197)	(0.858,4.3e-47)	(0.83,4.7e-274)	3p21.31
ENSG00000176095.10 (IP6K1)	(0.733,4.8e-27)	(0.823,1.3e-08)	(0.861,7.9e-210)	(0.87,1.0e-49)	(0.83,9.9e-266)	3p21.31
ENSG00000172046.17 (*USP19*)	(0.802,1.3e-35)	(0.925,1.1e-13)	(0.893,2.1e-246)	(0.87,8.8e-51)	(0.87,0.0e+00)	3p21.31
ENSG00000076201.13 (PTPN23)	(0.773,1.2e-31)	(0.856,8.6e-10)	(0.848,1.2e-196)	(0.846,1.5e-44)	(0.83,3.6e-274)	3p21.31

**Fig 4:**
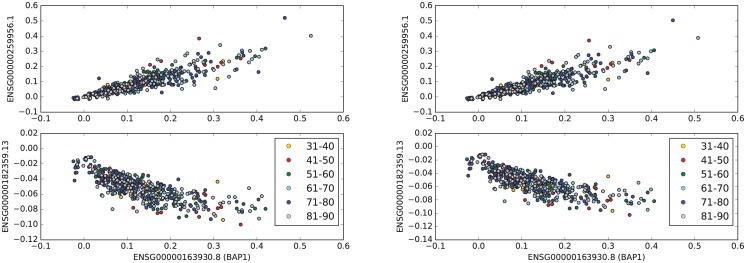
Highly correlated genes with *BAP1* in COAD. The top sub-figure shows the scatter plot of the values of *BAP1* and the gene, which has the maximum positive correlation with *BAP1* in the entire colon cancer data set. The bottom sub-figure shows the scatter plot of the values of *BAP1* and the gene, which has the maximum negative correlation with *BAP1* in all patients with colon adenocarcinoma. The right panel shows the exact same scatter plot as the left panel using n-normalization.

We also obtained the genes that were highly correlated with *BAP1* in samples obtained from primary tumor of patients with colon adenocarcinoma (See Table 6 and Fig 4). Interestingly, *USP19* and *RBM15B* were also highly correlated with *BAP1* expression in this data set.

**Table 6:** Highly correlated genes with *BAP1* in the entire COAD dataset.

transcript (gene)	(cor, p-value)	location
ENSG00000144028.13 (SNRNP200)	(0.802, 2.23e-107)	2q11.2
ENSG00000126062.3 (TMEM115)	(0.838, 5.45e-126)	3p21.31
ENSG00000259956.1 (RBM15B)	(0.875, 8.43e-150)	3p21.2
ENSG00000172046.17 (USP19)	(0.854, 2.15e-135)	3p21.31
ENSG00000070413.17 (DGCR2)	(0.823, 1.65e-117)	22q11.21
ENSG00000168066.19 (SF1)	(0.802, 3.84e-107)	11q13.1
ENSG00000114867.18 (EIF4G1)	(0.803, 1.74e-107)	3q27.1
ENSG00000163930.8 (BAP1)	(1.0, 0.00e+00)	3p21.1
ENSG00000169180.10 (XPO6)	(0.81, 4.12e-111)	16p12.1
ENSG00000076201.13 (PTPN23)	(0.838, 1.84e-125)	3p21.31
ENSG00000176095.10 (IP6K1)	(0.837, 3.53e-125)	3p21.31

The genes that were highly correlated with *BAP1* in the entire TCGA-BRCA, -UVM, and -COAD datasets have been presented in Tables 4, 5, and 6. There are three genes other than *BAP1*, ENSG00000259956.1 (*RBM15B*), ENSG00000172046.17 (*USP19*), and ENSG00000176095.10 (*IP6K1*), that belong to all three tables. Note that *IP6K1* was not in the list of highly correlated genes in the all four categories of breast cancer, because its correlation coefficient with *BAP1* in the black-alive category was 0.73, which was less than the threshold 0.8. However, its correlation coefficient with *BAP1* in the other three categories of breast cancer patients was greater than 0.8.

### No genes are highly negatively correlated with *BAP1*

**Fig 5:**
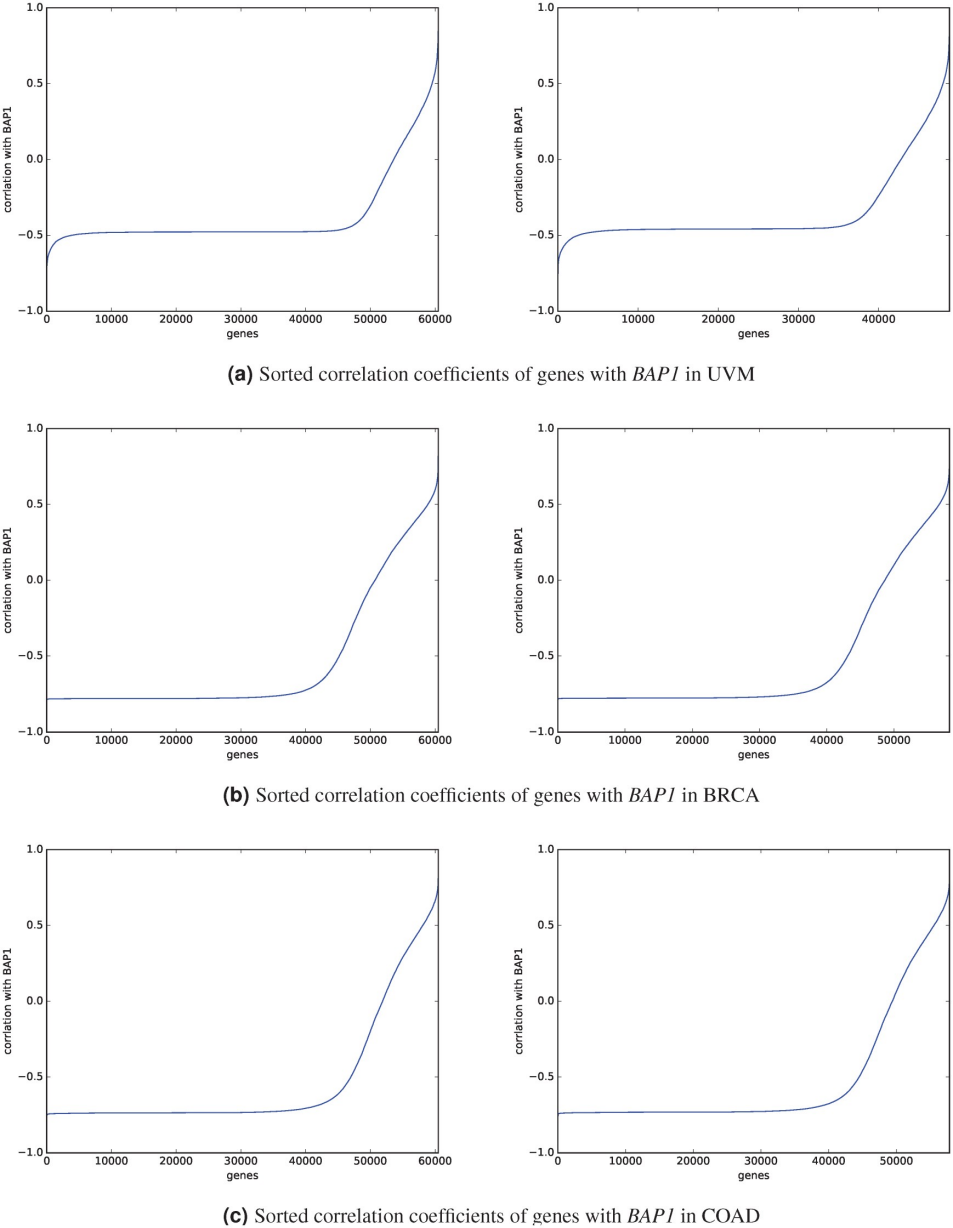
Sorted correlation coefficients of genes with *BAP1*. The left sub-figure shows the sorted correlation of all genes with *BAP1* in the dataset including genes with zero-values. The right sub-figure shows the results after using n-normalization (excluding genes with zero-values in the entire dataset).

Fig 5 shows the sorted correlation coefficients of all genes with *BAP1* in UM, breast, and colon cancer patients. Although most of genes are negatively correlated with *BAP1*, there is no gene, which is highly negatively correlated (correlation coefficient < −0.8) with *BAP1* in all data sets. Each category of breast cancer patients has some genes that are highly negatively correlated with *BAP1*, but there is no gene that is highly negatively correlated with *BAP1* in at least two categories. Furthermore, there are also no genes in the TCGA-UVM data set and also in the TCGA-COAD data set with correlation coefficient less than −0.8 with *BAP1*.

### Excluding genes with zero values did not change the results

We applied two normalization methods: 1) obtaining z-scores for each patient without excluding any genes and 2) eliminating genes with values of zero for all patients, then obtaining z-scores for each patient (for more details see Methods). These two normalization methods led to the same results.

## Discussion

Herein, we analyzed publicly available data sets from the TCGA and showed that *BAP1* plays a prognostic role in both UM and breast cancer. In contrast, survival was not associated with BAP1 expression in patients with colon cancer. There is a study showing that mutation of BAP1 only significantly affected overall survival in female patients with RCC [10]. In this project, which is the first study to our knowledge that evaluated *BAP1* associations with race and gender as well as age, we found that the *BAP1* expression does not correlate with race or gender in these data sets. However, the dead UM and breast cancer patients had lower levels of *BAP1* expression compared with surviving patients. Saliently, young dead patients with breast cancer had the lowest level of *BAP1*. This finding suggests that the level of *BAP1* could be a signature of aggressive tumors in UM and breast cancer, especially for young breast cancer patients. In contrast, survival was not associated with *BAP1* expression in patients with colon cancer.

**Fig 6:**
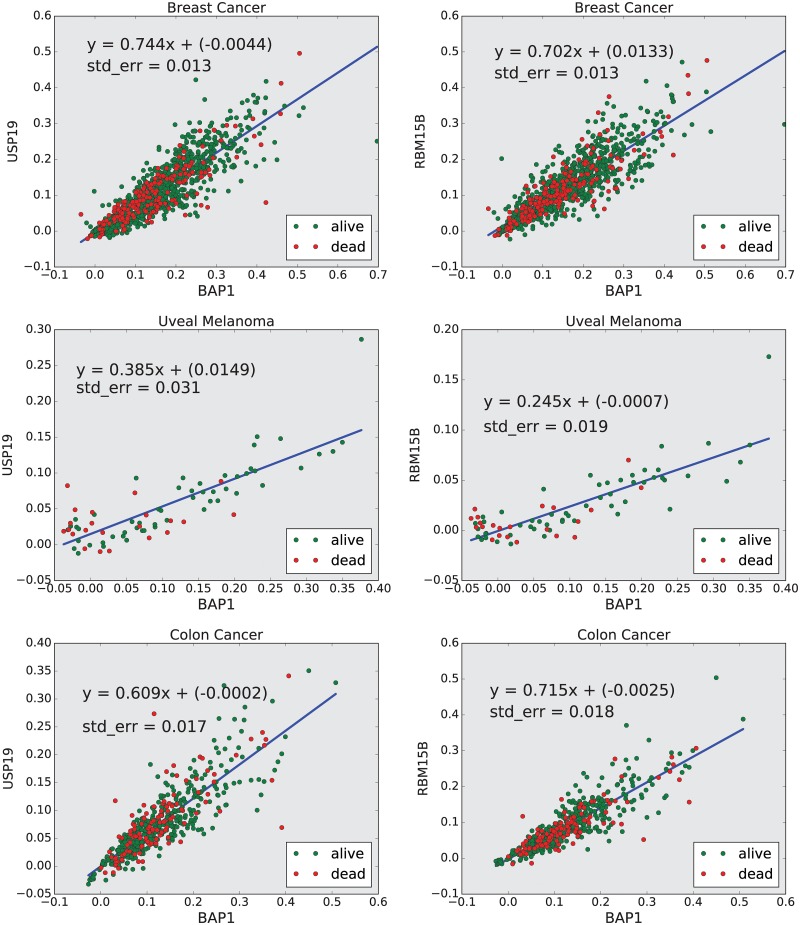
Regression analysis. This figure shows the linear function that predicts the normalized expression value of *USP19* and *RBM15B* from the normalized level of *BAP1*.

We discovered that there are three genes (*DHX30*, *USP19*, and *RBM15B*), which are highly positively correlated with *BAP1* in both black and white breast cancer patients. Among these three genes, two, *USP19* and *RBM15B* are also highly positively correlated with *BAP1* in UM. Importantly, *USP19* and *RBM15B* were also among 10 genes with a high positive correlation with *BAP1* expression in colon adenocarcinoma. To further learn these relationships, we performed a regression analysis to obtain the linear functions that predict the levels of USP19 and RBM15B from the level of BAP1. We found that the slopes of the lines modeling these linear relationships were higher in breast and colon cancers compared to uveal melanoma (See Fig 6).

The role of *BAP1* in breast cancer is still not clear. *BAP1* is located in the 3p21 chromosomal region, which is commonly deleted in breast cancer patients [11]. This suggests that *BAP1* could be an important driver for tumorigenesis in breast cancer. However, according to cBioPortal.org, there is a low frequency of *BAP1* somatic mutation (1.8%) in breast cancer. Also, a recent study shows that biallelic inactivation of *BAP1* is rare in breast cancer [12]. Our data show that *BAP1* expression is decreased in a subset of UM and breast cancer patients. This suggests that haploinsufficiency of *BAP1*, likely as part of the 3p21 chromosomal deletion, could be still important in the pathogenesis of breast cancers.

BAP1, which is a nuclear-localized tumor suppressor protein, has an ubiquitin carboxy-terminal hydrolase (UCH) domain resulting in its deubiquitinase activity. Although earlier studies suggested that BAP1 function is through modulation of the BRCA1, later studies showed that it is an independent tumor suppressor gene [10]. It has been shown that the deubiquitinating activity and nuclear localization are both required for *BAP1*-mediated tumor suppression [13]. The tumor suppressor function of *BAP1* is through its impact on cell cycle regulation, chromatin remodeling and DNA damage. BAP1, which is expressed in a temporal and spatial pattern during breast development and remodeling, binds to the BRCA1 RING finger motif, and pathogenic mutations in that domain negatively impact *BAP1* binding [14]. Additionally, a decrease in the level of BRCA1 protein has been observed in the malignant mesothelioma cells with *BAP1* deletion, while transduction of the mutants as well as WT *BAP1* results in an increase in the level of BRCA1 protein [15]. BAP1 enhances BRCA1-mediated suppression of cell proliferation through stabilizing BRCA1 [14]. However, BAP1 function is not through deubiquitination of the BRCA1/BARD1 complex.

USP19, which has been mostly studied in skeletal muscle atrophy, is a deubiquitinating enzyme [16]. It has been observed that the depletion of *USP19*, which is located in close proximity to BAP1 at 3p21.3 region, results in defects in cell cycle progression; *USP19*-depleted cells over-express *KPC1* and have reduced rates of growth [17]. It has been also reported that *USP19* inhibits *TNFα*-induced apoptosis via the stabilization of *c-IAP*s, which are ubiquitin ligases that regulate the stability of a variety of apoptotic and non-apoptotic proteins [18]. Importantly, a mutation in *USP19* and *LAMB2* has been observed in 12 patients with UM at high-risk of metastasis [19].

The RNA binding motif protein RBM15B (OTT3), which also located in the same chromosomal region 3p21, has been identified as a binding partner of the Epstein-Barr virus mRNA export factor [20] and found to be a cofactor of the nuclear export receptor *NXF1* [21]. Recently, it has been shown that RBM15B is located in a nuclear macromolecular complex through its direct binding to *CDK11*^*p*110^-cyclin L complexes and the splicing factor *9G8* [22]. Moreover, RBM15B is a functional competitor of the SR proteins, capable of inhibiting the formation of the spliceosomal E complex and antagonizing the positive effect of the *CDK11*^*p*110^-cyclin L2*α* complex on splicing [22]. Note that expression of the large *CDK11*^*p*110^ protein kinase isoforms is ubiquitous and constant throughout the cell cycle [23].

Of note, all highly positively correlated genes with *BAP1* in UM and breast cancer are located in chromosome 3. In metastatic UM, monosomy of chromosome 3 has been associated with short survival, compared to metastases with disomy or partial change in chromosome 3 [24]. Chromosome 3 has also been reported to play a role in prognosis of some other cancers, including in colon cancer and AML [25, 26]. Additionally, loss of chromosome 3 is well known as a poor prognostic factor in primary UM [5]. Importantly, allelic loss of several distinct regions on chromosome 3p including 3p25, 3p21.22, 3p21.3, 3p12.13 and 3p14 is one of the earliest and most frequent genomic abnormalities involved in a wide spectrum of major epithelial cancers including breast and colon [11, 27]. Chromosome 3p deletions occur in more than 80% of breast carcinomas [27]. This finding suggests that *USP19* and *RBM15B* could be in a functional pathway of *BAP1* or could be altered based on regional loss of chromosome 3p21.

In summary, our findings suggest that *BAP1* expression has prognostic significance in both UM and breast cancer. Future work to analyze *BAP1* expression in other cancers will be performed. An effort to develop targeted therapeutics for cells deficient in *BAP1* expression will be of great translational interest. The role of genes *RBM15B* and *USP19* may be important in *BAP1* function and give further insight into the mechanism of *BAP1* tumor suppression and additional targets for therapy.

## Materials and methods

We downloaded the TCGA transcriptome profiling data (HTSeq-FPKM-UQ files) of patients with UM, invasive breast carcinoma, and colon adenocarcinoma, from the GDC data portal (TCGA-UVM, TCGA-BRCA, and TCGA-CODA). For each of these cancer types, we downloaded the data sets data sets based on the age at diagnosis; we downloaded the data sets in the batches of 10 years interval starting from the minimum age at diagnosis. We divided the breast cancer data set into 2 main categories females and males. Since there were only 12 male patients, we only analyzed the data of female patients. We divided the female breast cancer patients to four sub-groups: black-alive (151 cases, 156 HTSeq-FPKM-UQ files), black-dead (29 cases, 31 HTSeq-FPKM-UQ files), white-alive (636 cases, 705 HTSeq-FPKM-UQ files), white-dead (112 cases, 159 HTSeq-FPKM-UQ files) (Table 2).

We divided UM and colon cancer patients into four sub-categories female-alive, female-dead, male-alive, and male-dead. Since the race was unknown for approximately half of these data sets, we only divided these two datasets based on gender, vital status, and age at diagnosis to several sub-categorizes. The TCGA-UVM data set includes 88 cases: 40 females and 44 males (Table 1). Since the number of cases in each category was small, we obtained the correlations with *BAP1* in the entire UVM data set rather than in each category. We collected the gene expression data from primary tumors of 456 patients with colon adenocarcinoma from TCGA-COAD project. This data set includes 478 HTSeq-FPKM-UQ files (Table 3).

In a recent study, it has been shown that gene expression profiles of patients have similar distributions, while the distribution of the expression value of each gene is different [28]. Furthermore, the genes’ expression level is a representation of interaction network of genes, if we normalize the value of each genes separately then we loose information. Thus, the best way to normalize the gene expression data sets from the primary tumors is normalizing each file (transcriptome profiling data of each patient), separately. Therefore, we standardized the gene expression profile of each patient separately to normalize the data. We applied two normalization methods: 1) obtaining z-scores for each patient without excluding any genes and 2) eliminating genes with values of zero for all patients, then obtaining z-scores for each patient. We called the second method n-normalization.

We performed several statistical analyses to see if the expression value of BAP1 is different in alive patients versus the dead ones. We applied the Mann-Whitney-Wilcoxon (MWW), also known as Mann-Whitney U-test, which is a nonparametric test of the null hypothesis that it is equally likely that a randomly selected value from one sample will be less than or greater than a randomly selected value from a second sample [29]. We have also used t-test to investigate whether the means of expression level of *BAP1* in two groups of alive and dead patients are statistically different from each other.

In order to obtain the genes, which are highly correlated with *BAP1*, first we normalized data by obtaining z-scores for each file independently. Then, we calculated the Pearson correlation between *BAP1* and all other genes. Specifically, we have a data set [*p*_1_, ⋯, *p*_*n*_], where *n* is the number of patients. Each *p*_*i*_ is a list of gene expression values, i.e. *p*_*i*_ = [*g*_1_⋯, *g*_*m*_], where each *g*_*j*_ is the expression value of transcript (gene) *j*, and *m* is the total number of transcripts (*m* = 60483). That means the data set is an *n* × *m*-dimensional matrix *D* = [*g*_*ij*_], where *g*_*ij*_ is the expression of transcript *j* in file (patient) *i*.

To normalize the data set, we obtained the z-score for each *p*_*i*_. Thus, we got a new matrix $\hat{D}=\left[{\hat{g}}_{ij}\right]$, where ${\hat{g}}_{ij}$’s are normalized values. Then, for each transcript *j*, we obtained Pearson correlation between the list $\left[{\hat{g}}_{ij},\;\;1\leq i\leq n\right]$ and the corresponding list for *BAP1* ($\left[{\hat{g}}_{iBAP1},\;\;1\leq i\leq n\right]$).
